# A Theoretical Perspective of the Photochemical Potential in the Spectral Performance of Photovoltaic Cells

**DOI:** 10.3390/e23050579

**Published:** 2021-05-08

**Authors:** Agustin Pérez-Madrid, Ivan Santamaría-Holek

**Affiliations:** 1 Departament de Física de la Matèria Condensada, Universitat de Barcelona, Martí i Franquès 1, 08028 Barcelona, Spain; agustiperezmadrid@ub.edu; 2 UMDI-Facultad de Ciencias, Universidad Nacional Autónoma de México Campus Juriquilla, Querétaro 76230, Mexico

**Keywords:** PVC, spectral response, quantum efficiency, spectral entropy production, photochemical potential, two-level atom model

## Abstract

We present a novel theoretical approach to the problem of light energy conversion in thermostated semiconductor junctions. Using the classical model of a two-level atom, we deduced formulas for the spectral response and the quantum efficiency in terms of the input photons’ non-zero chemical potential. We also calculated the spectral entropy production and the global efficiency parameter in the thermodynamic limit. The heat transferred to the thermostat results in a dissipative loss that appreciably controls the spectral quantities’ behavior and, therefore, the cell’s performance. The application of the obtained formulas to data extracted from photovoltaic cells enabled us to accurately interpolate experimental data for the spectral response and the quantum efficiency of cells based on Si-, GaAs, and CdTe, among others.

## 1. Introduction

In photovoltaic cells (PVCs), light absorption promotes the transference of electrons from the valence to the conduction bands, thus allowing electrical energy production from light. The efficiency with which this process occurs is crucial for these devices’ practical operation, and therefore, one of the fundamental aspects for theoretical investigations. The estimation of this efficiency follows the classical works by Schockley and Queisser [1]. This analysis tacitly assumes radiation-matter thermal equilibrium when employing the detailed balance relation. Posterior research improved this approach by considering that solar cells operate at steady-state conditions rather than at equilibrium; this was the case of the classical works by Wurfel [2] and Tiedje and coauthors [3].

References [4,5,6,7,8] provided classical contributions on this subject. The analysis of the efficiency during non-equilibrium operation starts by introducing the quasi-Fermi energy levels for the electrons and holes, from which the electrical potential generated by the charge separation originates. This electrical potential is the output voltage of the cell, and it is associated with the so-called internal chemical potential $\mu_{e}$, defined as the difference between the previously mentioned quasi-Fermi energy levels. These relations lead to the current-voltage relationship for the solar cell [3]. In its more simple form, these improvements are based on the two-level atom approach or can be reduced to it [9].

Recent theoretical approximations to the assessment of the spectral response and external quantum efficiency are diverse, (see, for instance, [10]), although the vast majority are based in computational programs solving the electrodynamic and electron and hole transport equations in semiconductor junctions under several assumptions; see, for instance, [11,12,13,14]. Analytical approaches are also reported in the phenomenological and quantum realms. In the first realm, the important contributions are associated with the introduction of light’s chemical potential, in a similar form as in the present work; see, for instance, the reports by Markvart and coworkers [15,16,17]. In the realm of dissipative quantum systems and quantum thermodynamics approach, the modeling is usually based on two-level atom systems for which generalized (LGKS) quantum master equations are used with the Jaynes–Cummings Hamiltonian with the Lindblad operator for the dissipative terms [18,19,20,21].

From a more practical perspective, PVCs’ performance is characterized by a series of quantities related to each other. The overall cell performance follows from the net power generated by the solar cell divided by the net input power provided by the incident radiation (the Sun). However, the spectral response, $S_{r}\left(\lambda \right)$, and the quantum efficiency $Q_{e}\left(\lambda \right)$, are the other two spectral quantities that measure the performance of PVC cells, providing precise information of this performance in terms of the frequency or wavelength. These two quantities are not independent. The relation between the quantum efficiency and the spectral response is:

(1) $$S_{r}\left(\lambda \right)=\frac{q\lambda }{hc}Q_{e}\left(\lambda \right)$$

In this work, we undertook the problem of calculating these two quantities from a two-level atom model and showed that they are proportional to the non-equilibrium photochemical potential of the incoming photons [17,22]. Our approach’s compelling perspective is to use these results to calculate mathematical relations for the spectral response and the quantum efficiency containing the peculiarities of the transition probabilities entering the equations that describe the population dynamics between states. We also discuss thermodynamic-related quantities like the spectral entropy production and the semiconductor’s efficiency for converting light input into an electrical current output. The merit of this work is that it shows that the key thermodynamic quantity in this whole situation is the photochemical potential. This quantity acts as the thermodynamic driving force, per wavelength band, yielding the entropy production of the cell and reducing its total efficiency.

Our analysis was based on a two-level atom model and did not consider recombination effects in terms of the classical view of electron-hole interaction. Nonetheless, we incorporated recombination processes due to radiative-induced processes and thermal coupling with the thermostat. As we show below, even in this case, the results obtained were auspicious since they allow one to reproduce quantitatively, with a fair degree of precision, experimental reports on the wavelength dependence of the spectral response and the quantum efficiency.

The two-level atom model is the most basic model describing appropriately the dynamics of the formation and recombination of minority carriers in semiconductors due to the incidence of light [9]. The model allows generalizations, like the inclusion of recombination effects and its extension to multi-state dynamics. Given its simplicity, it can easily be related to the non-equilibrium thermodynamics of photovoltaic cells. A direct correlation can be established between thermodynamic quantities and the quantum parameters entering the quasi-phenomenological Einstein model for the interaction between light and matter. From the quantum perspective, we used this level of approach because of the known difficulties of introducing dissipation due to the interaction with thermal reservoirs in the pure quantum approach [18,19,20,21].

The model is quasi-phenomenological as the Einstein coefficients $A_{nm}$ and $B_{mn}$ possess quantum microscopic bases, having its replica in alternative approaches just as in [18]. Similarly, the normalized state populations m(t) and n(t) correspond to the diagonal elements of the density matrix. Hence, the Einstein equations are neither more nor less than the diagonalized form of the quantum master equation.

The advantage of using this approach is that the statistical description of the dissipative processes can be directly related to the irreversible thermodynamics concepts and techniques. They differ from the traditional equilibrium approach because in addition to considering the unbalance of the temperatures between the light and the cell, the interaction with a thermostat to which a heat flow is continuously expelled during cell operation is also considered explicitly.

## 2. Radiative Energy-Exchange Out of Equilibrium: A Phenomenological Model

**Figure 1:**
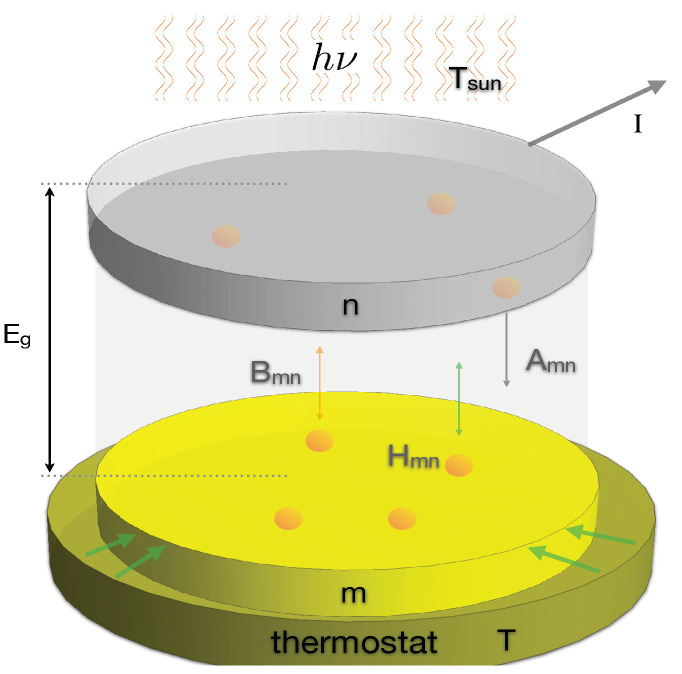
Schematic representation of the system considered. A junction in thermal contact with a thermostat at temperature *T*. The incoming radiation induces transitions of electrons from the valence (m) to the conduction (n) bands separated by the gap energy $E_{g}$ at the rates indicated.

Let us consider a semiconductor junction that exchanges energy with (i) the incoming radiation from the Sun and with (ii) a thermal bath with which energy is exchanged through heat conduction; see Figure 1.

The incoming light induces transitions of electrons from the valence to the conduction band, changing the semiconductor atoms’ energy distribution. In this representation, the dynamics of a two-level atom model copes with the essential physics when explicit electron-hole recombination is neglected [3,9]. This recombination could enter our model by including non-linear terms, i.e., defining reaction constants that depend on the occupation numbers of both states. However, this does not mean that two-level atom models lack recombination effects since transitions from the higher to the lower energy level are considered through radiative recombination, spontaneous emission, and thermal effects, all of them characterized by different rate constants. The transitions occur between the ground *m* and excited *n* bands of the semiconductor, having energies $E_{m}$ and $E_{n}$ that correspond to the quasi-Fermi energy levels of the valence and conduction bands [3]. We assumed that these transitions match stimulated and spontaneous emission and absorption of photons of frequency $h\nu =E_{g}=E_{n}-E_{m}$, where $E_{g}$ stands for the energy gap.

Mathematically, the model can be assembled with the help of the Einstein-like model [23,24] for which the evolution equation for the normalized number of atoms in the ground state, *m*, of the semiconductor materials is:

(2) $$\begin{array}{c} \frac{dm}{dt}=-\frac{dn}{dt}=-\left[B_{mn}u_{\nu }^{sun}+H_{mn}\right]m\;\;\;\;\;\;\;\;\;\;\;\;\;\;\;\;\;\;\;\\ \;\;\;\;\;\;\;\;\;\;\;\;\;\;\;\;\;\;\;\;\;\;\;+\left[B_{nm}u_{\nu }^{sun}+A_{nm}+H_{nm}\right]n, \end{array}$$

where it is essential to emphasize that, since the electromagnetic energy density producing the transitions has its origin in an external source, for example, the Sun, it approximately follows Planck’s radiation formula:

(3) $$u_{\nu }^{sun}=\frac{8\pi h\nu^{3}}{c^{3}}\frac{\epsilon_{\nu }^{sun}}{e^{h\nu /kT_{sun}}-1},$$

where $T_{sun}∼$ 5250–5600 K is the approximate temperature of the Sun and where we included the emissivity coefficient $\epsilon_{\nu }^{sun}$ that accounts for the deviation of the Sun’s emission with respect to the black body emission. Additionally, $B_{mn}\left(\nu \right)$ and $B_{nm}\left(\nu \right)$ are the transition probabilities per unit time due to the stimulated radiation process. These probabilities satisfy the detailed balance condition $g_{m}B_{mn}=g_{n}B_{nm}$, where $g_{i}$ are the degeneracies of states i=m,n. Additionally, the spontaneous transition rate from *n* to *m* is represented by $A_{nm}\left(\nu \right)$. Finally, the coefficients $H_{mn}$ and $H_{nm}$ measure the transition rates induced by the atoms’ thermal interaction with the thermostats. This last interaction is one of the main differences between our approach and the classical works reported in [1,2,3,4,5,6,7,8,15,17] and summarized in [25]. In these classical approaches, the assessment of the efficiency of the energy conversion is done by assuming a balance between absorbed and emitted radiation. In this approximation, thermal interaction with the heat reservoir is not explicitly considered in the microscopic mechanism as a possible factor promoting transitions. As will be shown below, the thermal contributions introduce corrections in the upward and backward transition rates, as well as the photochemical potential. Within the microscopic description based on a two-level system, the thermal factor was first considered in [22], where the radiative interaction between two black bodies was analyzed in approximated form.

The temperature difference between the Sun and the semiconductor acts as a drift that takes the semiconductor’s state away from equilibrium. Hence, it is convenient to rewrite Equation (2) in terms of the difference between electromagnetic energy densities: $\left(u_{\nu }^{sun}-e_{\nu }\right)$. Thus, by adding and subtracting terms in Equation (2), we may define the net radiative current:

(4) $$j\equiv -B_{mn}\left(u_{\nu }^{sun}-e_{\nu }\right)\left(n-m\right).$$

In this equation, the last term can be identified with the excess minority carrier in the semiconductor [26]. Using (4), we may write the more compact equations:

(5) $$dm/dt=-dn/dt=-j-k^{+}m+k^{-}n.$$

Equation (5) describes a first-order chemical reaction without the detailed balance, where $k^{+}$ and $k^{-}$ are the corresponding forward and reverse rate constants or probabilities per unit time of passing from ground to excited states and vice versa. These constants are given by:

(6) $$\begin{array}{c} k^{+}\equiv B_{mn}e_{\nu }+H_{mn},\;\;\;\;\;\;\;\;\;\;\;\;\;\;\;\;\;\;\;\;\;\;\;\;\;\\ k^{-}\equiv \left(g_{m}/g_{n}\right)B_{mn}e_{\nu }+A_{nm}+H_{nm}.\;\;\;\;\;\;\; \end{array}$$

It is worth mentioning that $k^{-}$ represents an “effective” recombination rate that incorporates spontaneous and radiation-induced recombination, as well as thermally activated recombination effects.

According to Equation (4), *j* is proportional to the net radiation received by the semiconductor from the Sun, which promotes the transference of atoms from the ground to their excited state.

### 2.1. Equilibrium

Thermal equilibrium is reached when the radiative current *j* and the time derivatives dm/dt=−dn/dt vanish. In this state, the equality of temperatures between the material, $T=T_{\nu }$, makes that Equation (5) reduce to:

(7) $$\frac{k^{+}}{k^{-}}=\frac{n^{eq}}{m^{eq}}.$$

The ratio of the equilibrium populations satisfies the canonical relation:

(8) $$\frac{n^{eq}}{m^{eq}}=\frac{g_{n}}{g_{m}}e^{-h\nu /kT}.$$

From Equations (6)–(8), it follows that

(9) $$e_{\nu }^{eq}=\frac{g_{n}A_{nm}}{g_{m}B_{mn}}\frac{1}{e^{h\nu /kT}-1}+\frac{H_{nm}-\left(g_{n}/g_{m}\right)H_{mn}e^{h\nu /kT}}{e^{h\nu /kT}-1},$$

where we used the detailed balance relation for the radiation-induced transition coefficients. In the black body ideal limit, the previous Equation (9) reduces to Planck’s radiation formula [24]:

(10) $$e_{\nu }^{eq}=u_{\nu }=\frac{8\pi h\nu^{3}}{c^{3}}\frac{1}{e^{h\nu /kT}-1}$$

when one takes into account the well-known relation $g_{n}A_{nm}/\left(g_{m}B_{mn}\right)=8\pi h\nu^{3}/c^{3}$ and the detailed balance relation $H_{nm}/H_{mn}=\left(g_{m}/g_{n}\right)e^{h\nu /kT}$ is satisfied, [24]. These results yield the following expression for the number of photons of frequency ν, $N_{\nu }$:

(11) $$N_{\nu }=\frac{1}{e^{h\nu /kT}-1},$$

### 2.2. Out of Equilibrium

Since $T_{sun}>>T$, then $j\propto u_{\nu }^{sun}-e_{\nu }\neq 0$, and the entire system is out of equilibrium since there is a net flux of the number of atoms that perform transitions from their ground states *m* to their excited states *n*. In the non-equilibrium stationary case, the time derivatives of the populations vanish, and Equation (5) reduces to:

(12) $$j=-k^{+}m^{st}+k^{-}n^{st},$$

where $n^{st}$ and $m^{st}$ are the populations in the non-equilibrium stationary state. Related to Equation (12), note that the left-hand side plays the role of a generating current (see Equation (4)), and the right-hand-side corresponds to the net recombination current in the classical view, in such a way that under steady-state illumination, both currents balance each other [25,26]. Using now Equations (4), (7), (8), and (12), we can obtain an expression for $n^{st}/m^{st}$:

(13) $$\frac{n^{st}}{m^{st}}=\frac{g_{n}}{g_{m}}\cdot \frac{1+B_{mn}\left(u_{\nu }^{sun}-e_{\nu }\right)/k^{+}}{1+B_{mn}\left(u_{\nu }^{sun}-e_{\nu }\right)/k^{-}}e^{-h\nu /kT}.$$

Notice that the involvement of the coefficient $B_{mn}$ in this last expressions came from Equation (4). The stationary radiative current *j* breaks the canonical balance due to the fact that it induces the passing of atoms from the lower to the upper energetic level, that is it produces a number of electrons in the conduction band. This effect does not modify the thermal equilibrium between the semiconductor and the thermostat.

The work that is necessary for the light to maintain the continuous production of free charge carriers, characterized by a stationary nonequilibrium number of atoms in the higher energy level, $n^{st}$, with degeneracy $g_{n}$, is accounted for through a macro-canonical correction associated with a free energy change per atom. Thus, we introduce the photochemical potential μ through the relation:

(14) $$\frac{n^{st}}{m^{st}}≃\frac{g_{n}}{g_{m}}e^{-h\nu /kT}e^{\mu /kT},$$

$m^{st}$ is the non-equilibrium number of atoms in the lower energy level with degeneracy $g_{m}$, corresponding to electrons in the valence band. Using Equations (13) and (14), one may obtain the explicit expression for:

(15) $$\begin{array}{c} \mu =kT\ln\left|\frac{1+\frac{B_{mn}}{k^{+}}\Delta u_{\nu }}{1+\frac{B_{mn}}{k_{i}^{-}}\Delta u_{\nu }}\right|. \end{array}$$

After performing some algebraic operations, the previous expression can be recasted in the more appealing form:

(16) $$\begin{array}{c} \mu =kT\ln\left|1+\frac{B_{mn}\left(\frac{1}{k^{+}}-\frac{1}{k^{-}}\right)\Delta u_{\nu }}{1+\frac{B_{mn}}{k_{i}^{-}}\Delta u_{\nu }}\right|. \end{array}$$

The last two formulas give the photochemical potential in terms of the difference of electromagnetic energy densities, $\Delta u_{\nu }=u_{\nu }^{sun}-e_{\nu }$, between the input radiation and the output emission by the semiconductor. It is therefore clear that this difference of electromagnetic energy at the surface of the semiconductor plays the role of a spectral drift, unbalancing the internal energy distribution of the semiconductor per frequency or wavelength band, owing to the production of an electrical current if the photochemical potential is large enough to overcome the potential barrier at the junction.

Equation (15) resembles previous relations obtained for the photochemical potential; see [15,17,22,25] and references therein. However, there are two main differences that should be emphasized. The first one is the way in which the energy input is incorporated through the difference of electromagnetic energy densities, $\Delta u_{\nu }=u_{\nu }^{sun}-e_{\nu }$, and the second and more important distinction is that Equation (15) incorporates the interaction with the thermal reservoir of the cell, through the quotients $B_{mn}/k^{+}$ and $B_{mn}/k^{-}$, that does not reduce to unity; see Equation (6).

## 3. Spectral Response, Quantum Efficiency, and Photochemical Potential

We want to emphasize here that Equations (4) and (15) constitute the fundamental results of this work, since they may be used to deduce important results, as well as to explore the influence of the drift $\Delta u_{\nu }$ on the electrical current produced by the junction.

### 3.1. The Current-Voltage Equation

Let us note now that Equation (4) gives the number of atoms per unit time passing from the valence to the conduction band after considering radiation and spontaneous and thermal recombination processes. Therefore, Equation (4) defines the net electric current produced by the junction under illumination. The light-generated current under short circuit conditions is:

(17) $$I_{n}=\frac{q\;j}{A},$$

where *q* is the charge of the electrons and *A* is the conduction cross-section area. Using the right-hand side of Equation (4), the light-generated current can be expressed in the form: $I_{n}=\frac{q}{A}\left(k^{-}n^{st}-k^{+}m^{st}\right)$. Now, after using Equation (7) and making some factorizations, the light-generated current becomes:

(18) $$I_{n}=\frac{q}{A}\frac{k^{-}}{m^{eq}}\left(n^{st}m^{eq}-m^{st}n^{eq}\right).$$

The factorization of the $m^{st}n^{eq}$ term again and the use of Equations (7), (8), and (15) enable us to obtain the following final relation between the light-generated current produced and the photochemical potential:

(19) $$I_{n}=I_{0}\left(e^{\mu /kT}-1\right).$$

where we introduced the notation $I_{0}\equiv qk^{+}m^{st}/A$. This definition indicates that the characteristic current $I_{0}$ is related to the free electrons’ joint production through light and thermal inputs. This formula goes parallel to the classical short-circuit current voltage [9]:

(20) $$I_{sc}={\bar{I}}_{0}\left(e^{qV_{oc}/kT}-1\right),$$

in which $I_{sc}$ is the short-circuit current and ${\bar{I}}_{0}$ is the reverse saturation current. The similitude between Equations (19) and (20) compels us to identify the photochemical potential with the voltage produced by the p-n junction:

(21) $$\mu =qV_{oc},$$

both being functions of the frequency. A similar result was obtained in [17] after analyzing the light-matter interaction in fluorescent solar collector. Additionally, it is interesting to notice that the equality (21) together with Equation (15) poses a novel way to relate the open cell voltage with the formation of minority carriers, which is similar to that reported in [26].

### 3.2. Spectral Response and Quantum Efficiency

The first result that follows from the previous analysis is the explicit expression of the spectral response $S_{r}$. This quantity is the light-generated current by the cell, $I_{n}$, divided by the power input of the incident light, $P_{i}$:

(22) $$S_{r}=\frac{I_{n}}{P_{i}}.$$

In a first approximation, the short-circuit current per photon is proportional to the open cell voltage, Equations (19) and (21), with μ provided by Equation (15), and to the intrinsic resistivity of the semiconductor ρ. The relation is:

(23) $$I_{n}=\frac{L}{\rho }\frac{\mu }{q},$$

where *L* is a characteristic length of the transport process, like the minority carrier diffusion length. The input power per photon by the incident light can be written as:

(24) $$P_{i}=h\nu \frac{c}{4L}.$$

Therefore, using Equations (23) and (24), the spectral response becomes:

(25) $$S_{r}\left(\lambda \right)=\frac{4L^{2}}{c\rho q}\frac{\lambda }{hc}\mu_{\lambda }.$$

Here, we switched from the frequency to the wavelength representation for convenience. According to Equation (1), the quantum efficiency is therefore given by:

(26) $$Q_{e}\left(\lambda \right)=\frac{4L^{2}}{c\rho q}\frac{\mu_{\lambda }}{q}.$$

Thus, Equations (25) and (26) emerge as fundamental spectral quantities providing precise information about the performance of p-n junctions and, in general, of PVCs. Here, Expression (26) indicates that the photochemical potential accounting for the unbalance induced by the light input power on the p-n junction is directly associated with the quantum efficiency of the cell, a spectral quantity that depends on the light absorption coefficient through the coefficients $B_{mn}$.

## 4. Applications

This section is devoted to using the relations obtained for the spectral response and the quantum efficiency. However, prior to the development of a direct comparison with the experimental data reported in the literature, it is convenient to discuss some crucial questions about the transition rates $B_{mn}$, $A_{nm}$, and $H_{mn}$ and their relation with the absorption coefficient.

### 4.1. Absorption Cross-Section and Spontaneous and Stimulated Transition Probabilities Per Unit Time

Einstein’s theory of radiation-matter interaction is a phenomenological theory embodied by Equation (2). Here, we added the thermostat’s interaction, not considered in the original model [24]. Hence, our approach depends on two inputs, $A_{nm}$ and $H_{mn}$. Non-relativistic quantum electrodynamics [27,28] establishes the microscopic foundations of Einstein’s phenomenological theory by providing the following expression for the spontaneous transition rate:

(27) $$A_{nm}∼\frac{|p_{nm}{|}^{2}}{3h\pi^{2}}\frac{\nu^{3}}{c^{3}},$$

where $p_{nm}=\langle n|er|m\rangle$ is the transition dipole matrix element due to the passing of electrons from the valence to conduction bands.

Furthermore, we took advantage of the relation between the transition dipole term and the absorption cross-section $\alpha_{\nu }$, and we write the stimulated transition probability per unit time in the form:

(28) $$B\left(\lambda \right)=\frac{\lambda^{3}}{8\pi h}A_{nm}\left(\lambda \right),$$

where we switched again from the frequency to the wavelength representation and dropped out the subindex notation for convenience. The relation between the spontaneous transition rate and the absorption cross-section is:

(29) $$A\left(\lambda \right)=\frac{8\pi }{\lambda^{2}}\alpha_{\lambda },$$

and the corresponding relation with the stimulated probability is $B_{mn}\left(\lambda \right)=\left(\lambda /h\right)\alpha_{\lambda }$, [29]. For the fittings below, we used the following mathematical relation for the absorption cross-section:

(30) $$\alpha \left(\lambda \right)=\frac{\lambda^{2}}{8\pi }A_{0}f\left(\lambda \right),$$

where $A_{0}$ is a characteristic transition frequency and f(λ) is an arbitrary function having the dimensions of frequency. The fits proceed by determining the constant $A_{0}$ and modeling the function f(λ).

The coefficients $H_{mn}$ and $H_{nm}$ measure the rates at which the electrons jump between the valence and conduction bands due to the thermal interaction with the heat bath. This thermal process suggests that $H_{mn}$ can follow Eyring’s formula:

(31) $$H_{mn}∼\frac{kT}{h}z_{nm}\left(T\right)e^{-E_{gap}/kT},$$

where $E_{gap}$ plays the role of an activation energy and $z_{nm}\left(T\right)=z_{n}/z_{m}$ with $z_{n}$ and $z_{m}$ the partition functions of the excited and basal states, respectively. For the sake of simplicity, in the following, this rate will be considered as a constant.

### 4.2. Data Fitting

The relations discussed in the previous subsection, together with the expression (15) for the photochemical potential, evidence that, for a given illumination spectrum, the wavelength behavior of the spectral response and, consequently, of the quantum efficiency become determined by the ratio between the thermal rate $H_{mn}$ and the spontaneous emission coefficient $A_{mn}$ or, equivalently, of the absorption cross-section $\alpha_{\lambda }$. We illustrate this latter point below by fitting experimental data [30,31,32].

**Figure 2:**
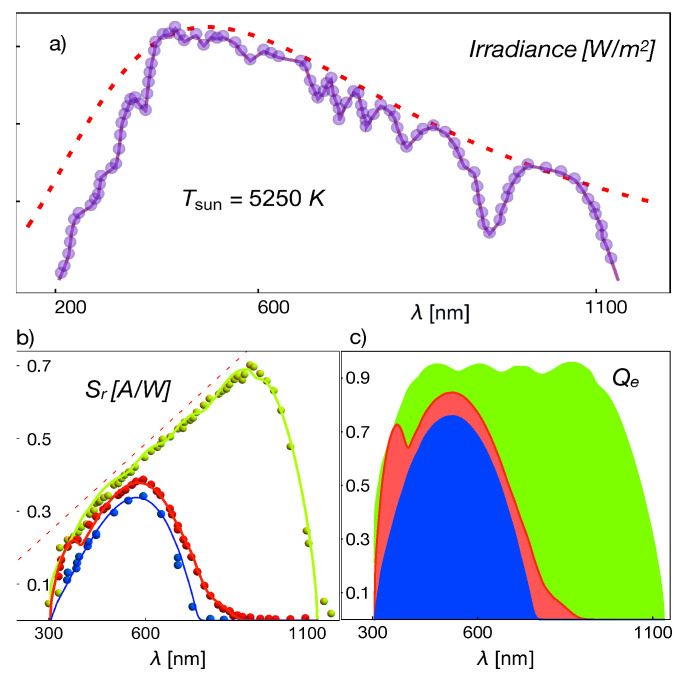
(**a**) Solar irradiance and interpolation of the data. (**b**) Spectral response, Equation (25) and (**c**) quantum efficiency, Equation (26), of different crystalline and amorphous Si-based cells [30] as a function of the wavelength for 1.5AMG. The green symbols and line correspond for the spectral response to AQ81/cr-Si, the red symbols and line to AQ82/cr-Si filtered, and the blue symbols and line to AQ83(4)/a-Si.

The data we used to compare with the theory were measured under the standard 1.5AM illumination protocol. Consequently, the incoming light spectrum should be approximated by Equation (3) and by adapting the wavelength dependence of the emissivity coefficient $\epsilon_{\lambda }^{sun}$. For this purpose, we introduced an interpolation function of the Sun’s irradiation data shown in Figure 2, for 1.5 AM illumination (symbols). The interpolation function used is the lighter purple line, whereas the red dashed line corresponds to the black body spectrum for the Sun’s approximated temperature $T_{sun}=5250$ K.

**Figure 3:**
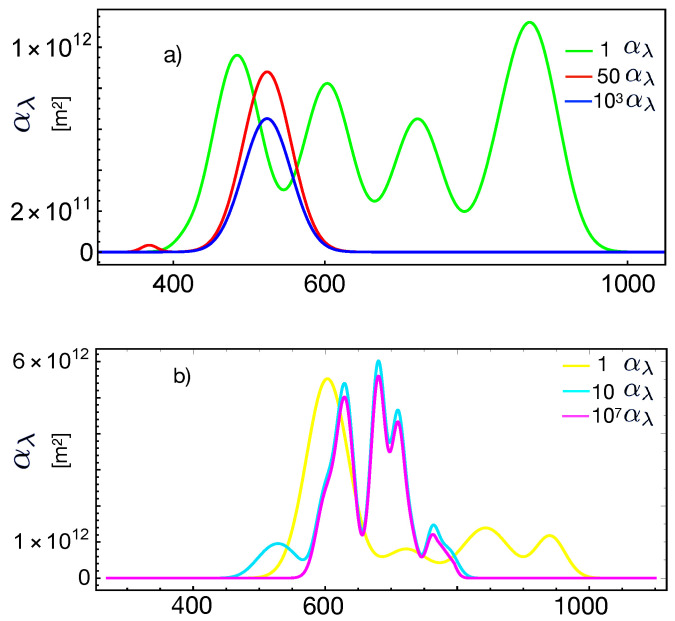
Spontaneous transitions rate $A_{mn}\left(\lambda \right)$ given by Equation (29) as a function of the wavelength. (**a**) The three coefficients used for the fits of Figure 2 with the same color key. (**b**) The three coefficients used for the fits of Figure 4 with the same color key.

Figure 2 shows the data (symbols) of the spectral response of crystalline and amorphous Si-based cells reported in [30], as well as the fit (lines) using Equation (25) with the corresponding absorption cross-section shown in Figure 3. The absorption cross-sections used were modeled, in a first approximation, by adding Gaussian functions with different amplitudes and variances. This figure also shows the external quantum efficiency, determined by Equation (26), and using the fits of the spectral response. The parameters used for the fit were $q=1.60\times 10^{-19}$ C, $\rho =2.5\times 10^{3}$ Ωm for the intrinsic resistivity. The characteristic length L=176 μm allowing a fit of the data with a 5% maximal error falls in the range reported in the literature for the electron diffusion length [33]. The values of the spontaneous emission coefficient $A_{0}$ and of the thermal rate $H_{mn}$ were: $A_{0}=1.14\times 10^{9}\;s^{-1}$ and $H_{mn}=3.5\times 10^{8}\;s^{-1}$.

**Figure 4:**
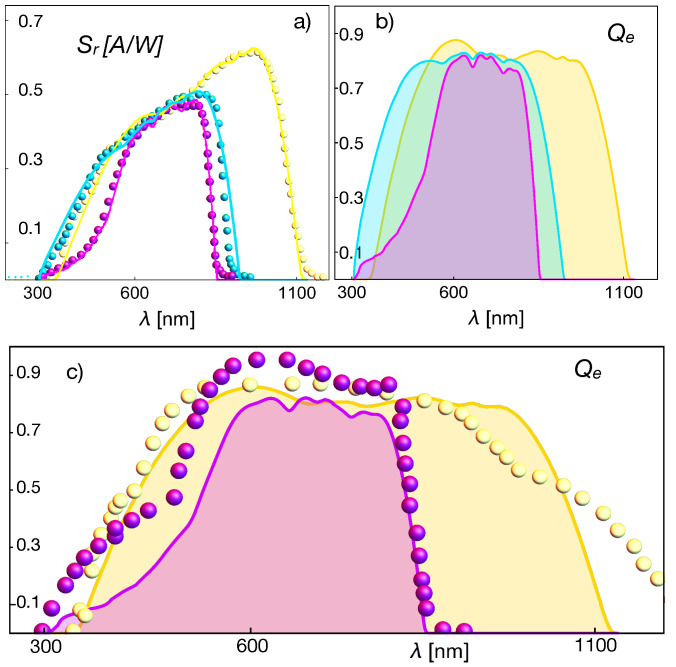
(**a**) Spectral response $S_{r}$, Equation (25), and (**b**) quantum efficiency, Equation (26), of different PVCs [31] as a function of the wavelength for 1.5AMG. The yellow symbols and lines correspond to a CIGS cell. The cyan symbols and lines correspond to GaAs-based and filtered cells and the magenta symbols and lines to a CdTe-based cell. (**c**) Comparison of the quantum efficiencies, inferred by using Equation (26), from the fit of CIGS and CdTe data from [31] with independent data from [32].

Figure 4 shows the data (symbols) of the spectral response of three cells based on different junction types (CIGS, GaAs, and CdTe) [31]. In a similar way as in Figure 2, also shown are the corresponding theoretical values of the quantum efficiency, inferred from Equation (26) and compared with the quantum efficiencies for CIGS (yellow) and CdTe (magenta), independently measured in [32]. The parameters used for the fit of the CIGS cell were: $\rho_{CIGS}=2.5\times 10^{3}$ Ωm for the intrinsic resistivity and the minority carrier characteristic length $L_{CIGS}=180$ μm. For the GaAs, we used $\rho_{GaAs}=1.08\times 10^{3}$ Ωm and $L_{GaAs}=90$ μm, and for the CdTe cell, we used $\rho_{CdTe}=1.5\times 10^{-2}$ Ωm and $L_{CdTe}=0.46$ μm. These values allowed the fitting of the data with an 8% maximal error and were in the range reported for the diffusion length reported in the literature [33,34]. The values used for the spontaneous emission coefficient and the thermal rate used in Figure 4 were: for CIGS, $A_{0}=1.14\times 10^{9}\;s^{-1}$ and $H_{mn}=3.5\times 10^{7}\;s^{-1}$; for GaAs, $A_{0}=5.4\times 10^{11}\;s^{-1}$ and $H_{mn}=35\;s^{-1}$; and for CdTe $A_{0}=3.1\times 10^{9}\;s^{-1}$ and $H_{mn}=8.0\times 10^{3}\;s^{-1}$. The fits were done using Mathematica after direct comparison between the formulae evaluation and the digitalized data. Data were digitalized using PlotDigitizer.

The inferred quantum efficiencies in Figure 2 and Figure 4 have the expected trend and values, thus indicating that the theoretical approach by Equations (25) and (26) is very promising for predicting these quantities if it is provided a precise model or data for the absorption cross-section. The relations also indicate that the spectral response data allow one to infer the absorption cross-section, as we proceeded in the present case.

**Figure 5:**
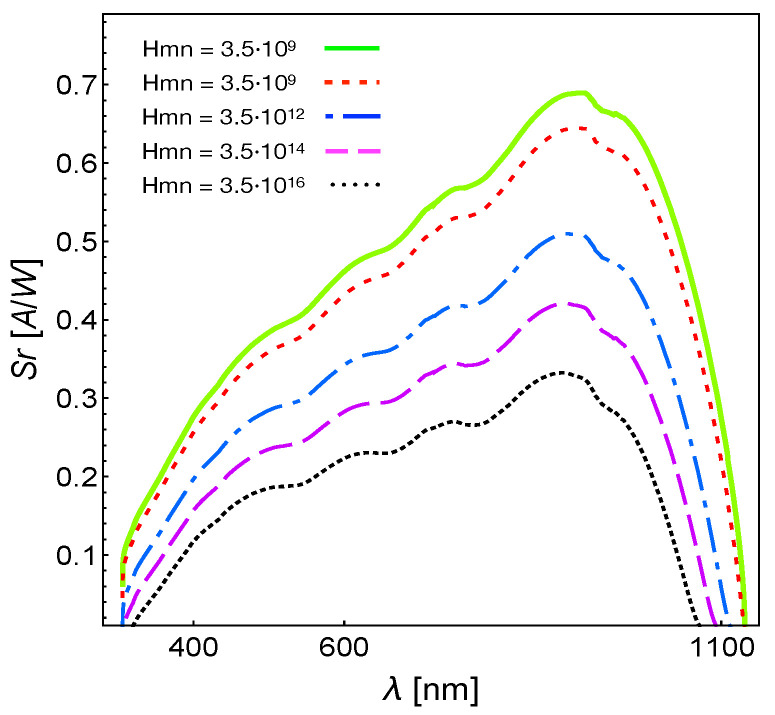
Behavior of the spectral response $S_{r}$, Equation (25), for different values of the ratio $A_{0}/H_{mn}$.

In Figure 5, we present the behavior of the spectral response $S_{r}$, Equation (25), for different values of the ratio $A_{0}/H_{mn}$. We kept the value of $A_{0}$ constant and took the case of AQ81/c-Si cells of Figure 2, to which corresponds the green line in Figure 5. The red dashed line follows by increasing the thermal rate by one order of magnitude, that is for $H_{mn}=3.5\times 10^{9}s^{-1}$. The blue dashed-dotted line corresponds to $H_{mn}=3.5\times 10^{12}s^{-1}$, the magenta long dashed line to $H_{mn}=3.5\times 10^{14}s^{-1}$ and, finally, the dotted black line to $H_{mn}=3.5\times 10^{16}s^{-1}$. The physical conclusion was that the intensity of the thermal interaction, characterized by an increasing value of the thermal rate $H_{mn}$, induces a significant reduction of the junction’s spectral response. It must be emphasized that Figure 5 shows that the spectral response depends dramatically on the coefficients $H_{mn}$. The ideal case would be for $H_{mn}=0$, and therefore, ignoring the parameters of the bath provides one with the maximum limits of the characteristic of photo-voltaic cells.

## 5. Entropy Production and Global Efficiency

This section evaluates the spectral entropy production of the semiconductor junction associated with the three different processes during its operation: current production, light absorption, and heat production.

The Gibbs entropy postulate [35] establishes that the entropy of the cell depends on the populations *n* and *m* in the form:

(32) $$S_{cell}=-k\;m\ln\left|\frac{m}{m^{eq}}\right|-k\;n\ln\left|\frac{n}{n^{eq}}\right|+S_{eq},$$

where $S_{eq}$ is the equilibrium entropy of the system and m(t) and n(t) evolve in time according to Equation (2). The time derivative of Equation (32) gives the total change of the entropy due to the three previously mentioned processes, since the evolution equation of the populations incorporates the corresponding energy (and entropy) exchanges due to the presence of the radiative and thermal transition rates.

Thus, performing the time derivative of Equation (32), using the fact that dm/dt=−dn/dt due to number conservation, and Equation (7), one can write the following expression for the entropy change per unit time:

(33) $$\frac{dS_{cell}}{dt}=k\;j\ln\left|\frac{k^{+}m}{k^{-}n}\right|+\left(k^{+}m-k^{-}n\right)\ln\left|\frac{k^{+}m}{k^{-}n}\right|.$$

The first term on the right-hand side of the last equation is the entropy exchange per unit of time (${\dot{\Phi }}_{cell}$) between the cell and the surroundings. This is due to the power input associated with the absorbed incoming light, which is proportional to *j*. The exchange of heat between the cell and the thermostat enters through the forward and backward rates $k^{+}$ and $k^{-}$. Recall that these rates depend on the thermal rates $H_{mn}$ and $H_{nm}$. The second term on the right-hand side is the spectral entropy production per unit time (${\dot{\Sigma }}_{cell}$) associated with the generation of free electrons due to the absorption of light and the heat exchange with the thermostat. As is well known, in the stationary state, the entropy exchanged with the surroundings compensates the entropy produced (${\dot{\Sigma }}_{cell}=-{\dot{\Phi }}_{cell}$), in such a way that the total entropy of the cell remains constant, $dS_{cell}/dt=0$. This condition implies Equation (5) and shows the consistency of our analysis.

Therefore, the entropy production per unit time, ${\dot{\Sigma }}_{cell}$, can be written in the form:

(34) $${\dot{\Sigma }}_{cell}=-k\;j\;\ln\left|\frac{k^{+}m}{k^{-}n}\right|.$$

**Figure 6:**
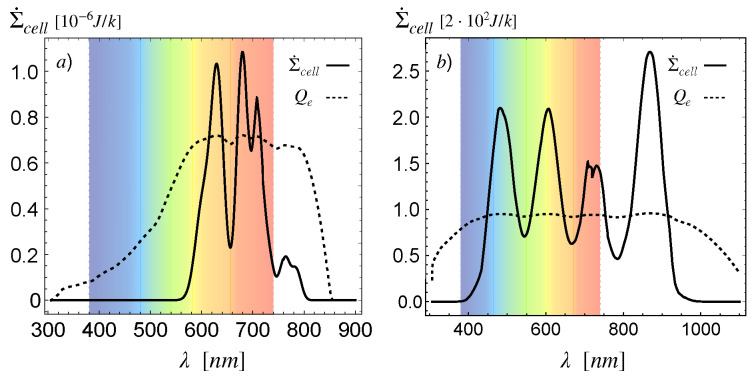
Quantum efficiency (26) and scaled spectral entropy production (35) of the (**a**) CdTe and the (**b**) cr-Si cells.

Using Equations (7) and (14) in order to rearrange the terms in the logarithm, it is possible to find:

(35) $${\dot{\Sigma }}_{cell}=\frac{j\;\mu_{\lambda }}{T},$$

which, in view of the definitions (17) and (21), is an alternative way to write the Joule heat effect, that is the power dissipated by an electric current. In Figure 6, we plot both the spectral entropy production given through Equation (35) and the quantum efficiency (26). The comparison shows that the light input energy is dissipated in generating minority carriers, showing the expected correlation between the quantum efficiency and the spectral entropy production.

Additionally, considering stationarity, the total entropy exchange ${\dot{\Phi }}_{cell}$ satisfies the equation:

(36) $${\dot{\Phi }}_{cell}=-{\dot{\Sigma }}_{cell}=k\;j\;\ln\left|\frac{k^{+}m}{k^{-}n}\right|.$$

This quantity can be divided into two parts, one associated with energy exchange between the cell and the radiation at temperature $T_{sun}$:

(37) $${\dot{\Phi }}_{cell}^{rad}=\frac{1}{T_{sun}}\frac{dU_{sun}}{dt}>0,$$

and the other one with the energy exchange between the cell and the thermostat at temperature *T*:

(38) $${\dot{\Phi }}_{cell}^{tst}=\frac{1}{T}\frac{dU_{tst}}{dt}<0,$$

where ${\dot{\Phi }}_{cell}={\dot{\Phi }}_{cell}^{rad}+{\dot{\Phi }}_{cell}^{tst}$, and the inequalities make reference to the fact that the thermal energy flow goes from the radiation field to the thermostat across the junction. Comparing Equations (37) and (38) with (36), one may infer that the stationary energy flow can be identified as:

(39) $$\frac{dU_{sun}}{dt}=-\frac{dU_{tst}}{dt}=h\nu \;j\equiv {\dot{Q}}_{\nu }.$$

Using the fact that $m^{st}-n^{st}=\tanh\left(\frac{\mu }{2kT}\right)$, since $m^{st}+n^{st}=1$, the explicit expression for the energy flow is:

(40) $${\dot{Q}}_{\nu }=B_{mn}\left(u_{\nu }^{sun}-e_{\nu }\right)\;h\nu \;\tanh\left(\frac{\mu }{2kT}\right).$$

This definition is compatible with Equation (4).

Using the relations between the induced transition coefficient $B_{mn}$ and the spontaneous emission $A_{nm}$, one may write Equation (40) in the form:

(41) $${\dot{Q}}_{\nu }=\frac{1}{\tau_{nm}}\left(N_{\nu }^{sun}-{\tilde{N}}_{\nu }\right)\;h\nu \;\tanh\left(\frac{\mu }{2kT}\right),$$

where $\tau_{nm}=1/A_{nm}$ is the average lifetime of the excited states. Additionally, the energy densities due to the Sun are simplified to $N_{\nu }^{sun}$, the Bose–Einstein distribution entering the Planck formula, Equation (11):

(42) $$N_{\nu }^{sun}=\frac{\epsilon_{\nu }^{sun}}{e^{h\nu /kT_{sun}}-1},$$

which is corrected by $\epsilon_{\nu }^{sun}$, the emissivity of the Sun consistent with the irradiance shown in Figure 2. It is worth recalling here that, in the quasi-equilibrium approximation, the definition (14) can be substituted into Equation (7) instead of $n^{eq}/m^{eq}$, thus leading to the Würfel–Planck distribution [6,36]:

(43) $${\tilde{e}}_{\nu }=\frac{8\pi h\nu^{3}}{c^{3}}\frac{1}{e^{(h\nu -\mu )/kT}-1}.$$

This last formula is used for calculating the backward emission flow by the semiconductor when the macroscopic radiation balance (40) is explicitly calculated. Equation (43) enables us to write the modified population number:

(44) $${\tilde{N}}_{\nu }=\frac{1}{e^{(h\nu -\mu )/kT}-1},$$

where the condition hν>μ should be obeyed. This condition is fulfilled in the whole range of wavelengths for the cells considered in this work and presented through Figure 2, Figure 3 and Figure 4. The representation of the quantum efficiency suggests that the photochemical potential in Equations (40) and (44) can be substituted by its maximum values $\mu ∼\mu_{max}$, which corresponds to the energy difference between the quasi-Fermi levels [9]. For instance, in the case of Figure 2, the value of the gap energy is about $E_{gap}≃1.7\times 10^{-19}$*J*, whereas for the green line, we have $\mu_{max}≃1.5\times 10^{-19}J$, for the red line $\mu_{max}≃1.3\times 10^{-19}J$, and finally, $\mu_{max}≃1.2\times 10^{-19}$ for the blue line. Therefore, Equation (44) takes the more usual form:

(45) $${\tilde{N}}_{\nu }=\frac{1}{e^{(h\nu -\mu_{max})/kT}-1}.$$

In view of the previous considerations, Equation (40) can be used for calculating the average efficiency of the cell if we first consider a proper distribution of excitation modes through their distribution of relaxation times for different frequencies or wavelengths, $\tau_{mn}\to \tau_{\nu }$, consistent with Equation (29). Introducing the normalized density of frequency modes $\rho_{\nu }$, for the solar cell power per unit surface, $P_{sc}$, we can write:

(46) $$P_{sc}=\tanh\left(\frac{\mu_{max}}{2kT}\right)\int_{E_{g}}^{\infty }h\nu \left(N_{\nu }^{sun}-{\tilde{N}}_{\nu }\right)\frac{\rho_{\nu }}{\tau_{\nu }}d\nu .$$

It must be emphasized that our approach can be extended to include a hierarchy of recombination processes [37] by introducing the appropriate distribution of frequency modes. As suggested in [38], this can be done by assuming $\rho_{\nu }∼1/\nu$. This definition of the solar cell power divided by the total solar input $P_{sun}=\sigma T_{s}^{4}$ gives the global efficiency of the cell:

(47) $$\eta =\frac{P_{sc}}{P_{sun}}=\frac{\tanh\left(\frac{\mu_{max}}{2kT}\right)}{\sigma T_{s}^{4}}\int_{E_{g}}^{\infty }h\nu \left(N_{\nu }^{sun}-{\tilde{N}}_{\nu }\right)\frac{\rho_{\nu }}{\tau_{\nu }}d\nu .$$

When $\rho_{\nu }/\tau_{\nu }=8\pi \nu^{2}/c^{2}$ and $\sigma =\pi^{2}k^{4}/\left(60c^{2}\hbar^{3}\right)$, then the expression obtained reduces to the classical one used in the literature for evaluating the solar cell efficiency, except by the correcting factor $\tanh\left(\frac{\mu_{max}}{2kT}\right)$, which originates from our treatment of the problem.

## 6. Conclusions

We attempted to formulate a novel theoretical approach to evaluate photovoltaic solar cells’ performance in this work. This performance was accounted for by the spectral response, the quantum efficiency, and the spectral entropy production at the microscopic level and in terms of the global efficiency parameter in the thermodynamic limit.

Our approach was based on the two-level atom model, which we modified by including the junction’s coupling with a thermostat that regulates its temperature. Considering the non-equilibrium nature of this energy exchange and conversion process, we adopted a stationary operation regime that breaks the detailed balance and substantially affects the results. The quantification of the non-equilibrium light absorption process was done through a stationary radiative current involving the difference between the input and output radiation from the cell and taking into account the crucial fact of the non-zero value of the chemical potential of photons in this non-equilibrated process. The combination of these two quantities and the appropriate definitions of the cell’s operation current and voltages allowed us to obtain an explicit expression for the spectral response of semiconductor junctions and therefore show that the quantum efficiency of the junction is proportional to the already mentioned photochemical potential. These relevant results allowed us to connect the spectral absorption cross-section with the spectral response and the cell’s quantum efficiency since the spontaneous and radiation-induced transition probabilities per unit time of the two-level atom model depend on this photochemical potential. Using the relationships we obtained, even for a rough model of the spectral absorption cross-section, we were able to perform good fits of the reported data of the spectral response and the quantum efficiency of different cells based on crystalline and amorphous Si, as well as for other junction compositions like GaAs and CdTe.

In addition, we showed that the efficiency of the junction’s energy conversion depends crucially on the ratio between the spontaneous to the thermally induced transition rates.

The theoretical approach presented here is parallel to the well-established solid-state description of the processes taking place in the energy conversion in semiconductor junctions. We believe that it is not a banal exercise. On the contrary, we think it contributes to the subject by clarifying how the coupling between light absorption and cell conversion efficiency depends on the photochemical potential. We consider that a possible limitation of our work consists of not having incorporated the multiple electron-hole recombination processes [37]. However, the model can be generalized in this way, a task that remains for future work.

## Data Availability

All data used were taken from published research and are cited in the captions of the figures. **©Mathematica programs (License) used** for evaluation of the formulae are available from the authors.

## Abbreviations

The following abbreviations are used in this manuscript:

PVC	Photovoltaic cell
cr-Si	Crystalline silicon
a-Si	Amorphous silicon
AQ81/c-Si	Crystalline silicon-based cell from [16]
AQ82/c-Si	Crystalline silicon-based mini-module cell from [16]
AQ83/c-Si	Crystalline silicon-based mini-module cell from [16]
AQ84/a-Si	Amorphous silicon-based mini-module cell from [16]
CIGS	Copper indium gallium selenide
GaAs	Gallium arsenide
CdTe	Cadmium telluride
